# A little elastic for a better performance: kinesiotaping of the motor effector modulates neural mechanisms for rhythmic movements

**DOI:** 10.3389/fnsys.2014.00181

**Published:** 2014-09-25

**Authors:** Riccardo Bravi, Eros Quarta, Erez J. Cohen, Anna Gottard, Diego Minciacchi

**Affiliations:** ^1^Department of Experimental and Clinical Medicine, University of FlorenceFlorence, Italy; ^2^Department of Statistics, Informatics, Applications, University of FlorenceFlorence, Italy

**Keywords:** sensory-motor integration, timing, isochronous movements, auditory imagery, music

## Abstract

A rhythmic motor performance is brought about by an integration of timing information with movements. Investigations on the millisecond time scale distinguish two forms of time control, event-based timing and emergent timing. While event-based timing asserts the existence of a central internal timekeeper for the control of repetitive movements, the emergent timing perspective claims that timing emerges from dynamic control of nontemporal movements parameters. We have recently demonstrated that the precision of an isochronous performance, defined as performance of repeated movements having a uniform duration, was insensible to auditory stimuli of various characteristics (Bravi et al., 2014). Such finding has led us to investigate whether the application of an elastic therapeutic tape (Kinesio® Tex taping; KTT) used for treating athletic injuries and a variety of physical disorders, is able to reduce the timing variability of repetitive rhythmic movement. Young healthy subjects, tested with and without KTT, have participated in sessions in which sets of repeated isochronous wrist's flexion-extensions (IWFEs) were performed under various auditory conditions and during their recall. Kinematics was recorded and temporal parameters were extracted and analyzed. Our results show that the application of KTT decreases the variability of rhythmic movements by a 2-fold effect: on the one hand KTT provides extra proprioceptive information activating cutaneous mechanoreceptors, on the other KTT biases toward the emergent timing thus modulating the processes for rhythmic movements. Therefore, KTT appears able to render movements less audio dependent by relieving, at least partially, the central structures from time control and making available more resources for an augmented performance.

## Introduction

Considerable evidence suggest that the study of human motor rhythmic performance synchronized to auditory stimuli is useful in unraveling neural aspects of action timing. The effects of different types of audio information (i.e., metronome or music) on motor performance have been extensively investigated in studies of finger tapping (Repp, 1999a,b; del Olmo and Cudeiro, 2005) and of gait (see Wittwer et al., 2013). The interest for the effects of paced auditory stimuli on rhythmic activities stems from the proven notion that this design of sensorimotor integration is shown to be an effective tool for gross motor rehabilitation in neurological diseases by improving spatial-temporal parameters and decreasing temporal variability (Thaut et al., 1996; McIntosh et al., 1997; del Olmo and Cudeiro, 2005; del Olmo et al., 2006; Hausdorff et al., 2007; Arias and Cudeiro, 2008; de Dreu et al., 2012).

In a recent study we showed how do auditory stimuli with distinct characteristics, streams of clicks or excerpts of music, influence the precision of repeated isochronous wrist's flexion-extensions (IWFEs), performed with no direct surface opposition and minimizing visual information (Bravi et al., 2014). Some reports have described a reduction of temporal variability for rhythmic movements in the presence of paced auditory stimuli. Our data, however, displayed clearly that when both tactile and visual information are minimized, the listening alone to paced auditory stimuli does not improve the precision of an isochronous performance. Also, in a previous study on timing of rhythmic movements a lack of reduction in temporal variability was observed during paced tapping when compared to unpaced tapping (Schlerf et al., 2007). These findings have prompted us to investigate other tools that might augment the precision of rhythmic movement.

In addition, investigations on the millisecond time scale distinguish two forms of time control, explicit timing and implicit timing, later renamed as event-based timing and emergent timing, respectively, (Spencer and Ivry, 2005). Event-based timing has evolved from the information processing approach, asserting the existence of internal timekeepers for motor control, and is well represented by the two-level model proposed by Wing and Kristofferson (1973a,b). According to the Wing and Kristofferson model, temporal precision in a self-paced tapping is limited by variability in the central timekeeper and by variability arising from the peripheral motor system (Semjen et al., 2000). The emergent timing perspective is derived from the dynamic systems theory, claiming that there are no representational clocks (Kugler et al., 1980; Kelso, 1995) and that timing emerges from dynamic control of nontemporal movements parameters (Schoner, 2002; Huys et al., 2008) such as stiffness which is playing a major role for determining movement frequency (Turvey, 1977; Delignières et al., 2004). For the study of timing, the task most widely employed was the performance of repeated movements. Among the rhythmic motor tasks, tapping is thought to be under the control of the event-based timing while circle drawing is thought to be under the control of the emergent timing (Zelaznik et al., 2002). Air tapping, a motor task performed in the air with no surface contact, represents a more recent approach for the study of timing. This motor task possesses specific characteristics of both the event-based and emergently motor rhythmic tasks due to the presence of salient motor events and smooth effector trajectories (Delignières and Torre, 2011).

The participation of the cerebellum in timing was once a controversial issue (Leiner et al., 1993; Rao et al., 2001). To date, the cerebellar timing hypothesis, first formulated by Keele and Ivry (1990), is widely accepted. According to this hypothesis, the cerebellum functions as an internal timing device in the milliseconds range for both motor and non-motor processes (Ivry and Spencer, 2004). In particular, the involvement of the inferior olive and the climbing fiber system in timing was shown to take part in encoding temporal information independent of motor behavior (Xu et al., 2006). Investigations using rhythmic tapping tasks have provided evidences to support this hypothesis. In a study on individuals with unilateral cerebellar lesions, participants were asked to perform timed tapping, intermittent circle drawing, and continuous circle drawing tasks. Performances were impaired only when tasks were executed with the ipsilesional hand and involved movements theorized to require event-based but not emergent timing (Spencer et al., 2003).

The timing mode of rhythmic movements can change over time within a single task (Studenka and Zelaznik, 2008; Zelaznik and Rosenbaum, 2010; Delignières and Torre, 2011). The discrete or continuous character of movements performed in the rhythmic tasks is considered to be a key factor for the involvement of the event-based or the emergent timing mode (Robertson et al., 1999; Huys et al., 2008). In addition, neurophysiological and neuroanatomical studies provided bases to suggest that neural circuits are not completely independent of the timing functions. For discrete movements, it is proposed that explicit processes for timing arise as coordinated activity in the core striatal and olivo-cerebellar networks that are interconnected, with each other and with the cerebral cortex, through multiple synaptic pathways (Spencer et al., 2007; Teki et al., 2012). Conversely continuous movements, by nature, lack an event structure. For these movements timing may be achieved through the control of a secondary variable, such as angular velocity, which does not involve the cerebellum.

Kinesio® Tex Tape is an elastic cotton strip with an acrylic adhesive developed by Japanese chiropractor, Dr. Kenso Kase, in the 1970's (Kase et al., 2013). Kinesio® Tex taping (KTT) is a kinesthetic method commonly used in clinical practice for treating athletic injuries and a variety of physical disorders, with the purpose of mimicking the thickness and flexibility of the skin (Morris et al., 2013). It is claimed that the application of KTT causes micro convolutions, or folds, in the skin; it brings about a lifting of the skin away from the tissue beneath, favoring the release of pressure from tender tissues underneath and providing space for lymphatic fluid movement (Morris et al., 2013).

It was proposed that KTT is able to enhance somatosensory inputs and influence proprioception through stimulation of cutaneous mechanoreceptors (Callaghan et al., 2002; Kneeshaw, 2002; Halseth et al., 2004). We have, therefore, chosen KTT as a tool for possibly augmenting the precision of rhythmic movements. While little is known about the potential proprioceptive effect of KTT, the stimulation of cutaneous mechanoreceptors is believed to be induced by the pressure and stretching effect provoked by KTT application on the skin (Grigg, 1994).

In the current study we investigate whether the application of KTT on skin is able to reduce the timing variability of repetitive rhythmic movements. We also attempt to understand whether a causal relationship subsists between such reduction and functional augmentation of central structures involved, brought about by relieving, at least partially, these structures from time control.

We thus performed an experiment in which subjects, tested with and without KTT, participated in sessions in which sets of repeated IWFEs were performed under different conditions (i.e., without audio information, with paced audio in the form of clicks or music, and in the recall conditions; see Bravi et al., 2014). Our IWFEs performed with no direct surface opposition and while minimizing visual information, could potentially employ, as air finger tapping movements, both event-based and emergent timing processes. Participants were not instructed about the way to perform the IWFEs since it was showed that in the absence of specific instructions, the production of movements with no contact surface elicits alternatively different timing modes (Delignières and Torre, 2011).

We hypothesize that KTT, due to its aforementioned characteristics, is able to cause an improvement of the wrist joint proprioception due to augmented afferent input via the stimulation of cutaneous mechanoreceptors (Riemann and Lephart, 2002). The improvement in wrist joint proprioception would stabilize the motor effector during the performance, causing the IWFEs to be less discrete and thus smoother. Consequently this would entail the shifting from an event-based to emergent process to control the production of the IWFEs (Spencer et al., 2007; Huys et al., 2008). Therefore, by relieving, at least partially, the central structures from timing control, KTT would release resources and allow for a net augmentation of the central efficiency and, consequently, reduce the timing variability of IWFEs.

## Materials and methods

### Participants

A group of 25 right-handed volunteers (21 males, ages 20–27, mean 24.1 ± 2.4 years; 4 females, ages 18–27, mean 23.0 ± 2.7 years) took part in the recording sessions. All participants were naive to the purpose of the study; they were neither musically trained nor listeners of a classical music repertoire, and reported no auditory, motor, or other neurological impairments. The experimental protocol conformed to the requirements of the Federal Policy for the Protection of Human Subjects (U.S. Office of Science and Technology Policy) and of the Declaration of Helsinki, and has been approved by the Research Ethics Board of our Institution (Local Ethics Committee, Azienda Ospedaliero Universitaria Careggi, Florence, Italy). All participants provided an informed consent in written form.

### Set up

**Figure 1:**
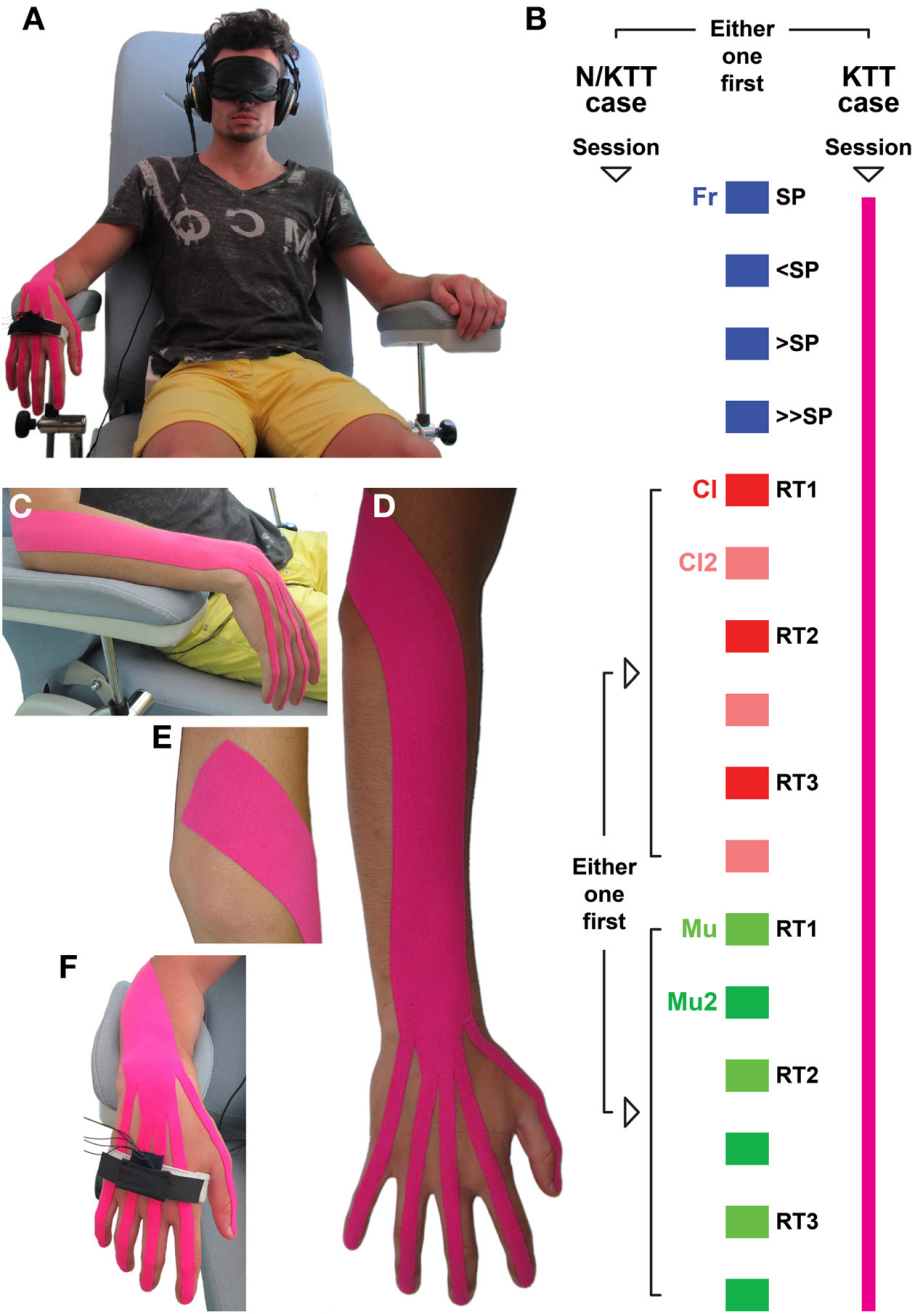
**(A)** The subject is seated on a chair with armrests, wearing an eye-mask and headphones. The triaxial accelerometer is placed over the 2nd–3rd metacarpal bones. Subject is asked to perform sets of IWFEs. **(B)** The two sessions, one with N/KTT and one with KTT (color-coded in pink) are designed to record first IWFEs in condition free from auditory information (in the absence of auditory information, free = Fr, self-paced = SP; slower than SP = <SP; faster than SP = >SP; faster than >SP = >>SP) and then in conditions related to clicks (during the listening of clicks = Cl; recall after 2 min from the end of the Cl condition = Cl2; one block consisting of three pairs of Cl and Cl2 sets performed at three different reference tempi: RT1, RT2, and RT3) followed by those related to music (during the listening of music = Mu; recall after 2 min from the end of the Mu condition = Mu2; one block consisting of three pairs Mu and Mu2 sets performed at three different reference tempi: RT1, RT2, and RT3), or vice versa. In this and in the following figures the conditions and associated results are color-coded. **(C)** KTT was placed between the lateral epicondyle of the humerus and phalanges, at the position of maximum flexion. **(D)** KTT was applied over the extensor carpi ulnaris, extensor carpi radialis longus and brevis, extensor digitorum, extensor indicis, extensor digiti minimi, and extensor pollicis longus. **(E)** Proximally, KTT was placed from 5 cm proximal to muscles insertion. **(F)** Each branch of tape was applied down on the back of phalanx until the nail. As in the N/KTT set up, the accelerometer was placed on the dorsal aspect of the hand, over the proximal part of the 2nd–3rd metacarpal bones, into a pocket kept in position by an elastic band and secured by a Velcro strap.

Each subject was tested individually, sitting upright on a chair with the legs comfortably positioned on leg-rest (Figure 1A). The participant's right forearm was placed on the armrest, in a relaxed horizontal position. The wrist and hand of the subject were free to move in mid-air with no direct opposition, thus minimizing tactile information. In order to reduce possible interference of visual information, the subject was requested to wear a blindfold. Environmental noise was reduced by the use of headphones (K 240 Studio, AKG Acoustics GmbH, Wien, AT), which were also used for the clean delivery of audio information. This setup was designed to minimize all information that is known to influence motor strategies (i.e., visual, tactile, and environmental noise) and that may result in a different motor performance (Bove et al., 2009; Saijo and Gomi, 2010). A triaxial accelerometer (ADXL330, Analog Devices Inc., Norwood, MA 02062) was placed on the dorsal aspect of the hand, over the proximal part of the 2nd–3rd metacarpal bones. The sensor, placed in a small pocket, was kept in position by an elastic band and secured by a Velcro strap. Sensor output was acquired and digitized at 200 Hz through PCI-6071E (12-Bit E Series Multifunction DAQ, National Instruments, Austin, TX, USA).

### Stimuli

Audio stimuli were paced streams of clicks and excerpts of music. Music excerpts of 60 s duration were extracted from original professional productions. Selected fragments had a simple and explicit rhythmic structure and a tempo that remained constant throughout time. Music fragments were taken from musical styles popular among young people: rock, techno, dance, trance, hard rock, and film music. Three musical experts (professionals with musical experience) were asked to verify independently the tempo of the excerpts in order to be as accurate and sure as possible in the choice of unambiguous paced excerpts. It is known that the determination of perceptual musical tempo can be ambiguous (McKinney and Moelants, 2007) and that algorithms for music tempo extraction have several limitations (McKinney et al., 2007), although recent effort has been put into methods to possibly improve the evaluation metrics used for automatic tempo estimation (Levy, 2011). Only fragments for which the experts collectively agreed of having a single musical tempo were selected. All chosen fragments had small tempo variations due to live performance. Therefore, the tempo for each fragment was analyzed, beat-mapped, and set to fixed beats per minute (bpm, used as a measure of tempo in music) using the softwares Jackson (http://vanaeken.com/; accuracy is within a 0.001 bpm margin) and Audacity (GNU/GPL, http://audacity.sourceforge.net/). Our time manipulations were all well below 5% deviation of the mean music tempo (professional performances have minimal deviations from mean tempo; Dannenberg and Mohan, 2011) and thus below the detection threshold for temporal changes in music (Ellis, 1991; Madison and Paulin, 2010). We didn't use fragments likely to be familiar to the listeners since it has been shown that high familiarity with a song installs an accurate long-term memory of its tempo (Levitin and Cook, 1996; Quinn and Watt, 2006). Streams of clicks of 60 s duration were produced using the audio editor Audacity, via the Generate Click Track function. The duration of an individual click was set to 10 ms and the click sound was set to white noise. The music and clicks stimuli were set to eight reference tempi ranging from 64 to 176 bpm (937.21 to 340.95 ms, Table 1). The same eight tempi were chosen for both streams of clicks and excerpts of music. Audio files were all normalized to −0.3 dB as the highest peak and stored as waveform (waveform audio file; 44.1 kHz; 16-bit depth) using Audacity. Fade in and fade out (10 ms) were placed at the beginning and end of each file. Audio stimuli were presented binaurally to the subjects through headphones (Figure 1A).

**Table 1:** **Musical names, ranking, and tempo equivalences between ranges of movement durations in Hertz and milliseconds**.

**Musical names**	**Rank**	**Tempo equivalences**					
		**Ranges (Hz)**			**Ranges (ms)**		
		**Reference**	**From**	**To**	**Reference**	**From**	**To**
Adagio	1	1.067	1.017	1.133	937.21	983.28	882.61
Andante	2	1.333	1.283	1.400	750.19	779.42	714.29
	3	1.600	1.550	1.667	625.00	645.16	599.88
Moderato	4	1.867	1.817	1.933	535.62	550.36	517.33
	5	2.133	2.083	2.200	468.82	480.08	454.55
Allegro	6	2.400	2.350	2.467	416.67	425.53	405.35
	7	2.700	2.617	2.800	370.37	382.12	357.14
Presto	8	2.933	2.817	3.000	340.95	354.99	333.33

### Data format

Subjects were asked to perform sets of IWFEs and kinematic parameters were evaluated. Data from the sensor were stored and an off-line analysis of raw data from the triaxial accelerometer was implemented. The signal extracted from the accelerometer presented a minimum when the wrist reached the maximum flexion and a maximum when it reached the maximum extension. The duration of a single wrist's flexion-extension (i.e., IWFE duration) was calculated as the difference between two consecutive flexion-extension minima (custom software developed in Matlab®). To avoid initial and final transients, the first and last 5 s of each recording were excluded from analysis (Repp, 2005, 2011). Considering that tempi of the recorded IWFEs sets were remarkably variable, giving rise to a quantitative redundancy of tempi obtained in overall, we have decided to rank them as ranges (Table 1). IWFEs sets were then assigned to a defined range according to their tempo.

### Session

Each subject had participated in two sessions, one with no KTT (N/KTT) and one with KTT (Figure 1B), that were performed at a week's distance. The order in which subjects performed the N/KTT or KTT sessions were set to obtain equivalent number of subjects that played first one or the other. The KTT session started approximately 10 min after mounting the tape since this interval is needed to overcome perception of tape on the skin (Kase et al., 2013). In each session the subject performed 16 sets of repeated IWFEs under various conditions. A single session was comprised of a baseline recording (4 sets) and 2 blocks (12 sets). In a single session the performance of the IWFEs sets was executed under five conditions: in absence of auditory information (free, Fr), while listening to a stream of clicks (Cl), after 2 min from the end of the Cl condition (Cl2), while listening to an excerpt of music (Mu), after 2 min from the end of the Mu condition (Mu2). Sets of IWFEs were recorded in all conditions. For each block IWFEs sets were performed using one type of auditory stimulus (Cl or Mu). One block consisted of three pairs of Cl and Cl2 sets, or Mu and Mu2 sets, performed at three different reference tempi, for the auditory Cl or Mu sets; the same tempi were then used for the auditory sets of the second block (Figure 1B). The order in which the clicks or music stimuli were presented was set to obtain equivalent number of subjects that received first one or the other.

The subject was instructed to restrain from listening to music during the day of the session. Each session began with instructions on how the sets of wrist's flexion-extensions were to be performed, followed by a short practice test. During the practice test, IWFEs were recorded and evaluated to assess whether the instructions were understood; after the practice test, the subject was asked if he/she felt comfortable with the task. Recordings from the practice test were not used for further analyses. The session started with recording in the Fr condition, which consisted of four IWFEs sets. In the first set, the subject was asked to perform IWFEs in a self-paced manner. In the second set, the subject was asked to perform IWFEs slower than those of the first set. In the third and fourth sets, the subject was asked to perform IWFEs faster than first recording and then faster than the third recording. The subject was then instructed to perform a set of repetitive IWFEs while listening to clicks or music at a similar pace concurrently with the tempo of the delivered audio (i.e., maintaining an in-phase 1:1 relationship between repeated wrist's flexion-extensions and the tempo of the audio, conditions Cl or Mu). Then, instructions to subject for IWFEs performances in recalls were of the kind: “now, try to hear in your head the auditory stimulus just heard” (immediately after the end of the stimulus, see Zatorre and Halpern, 2005). After a 2 min break, the subject was asked to perform IWFEs as accurately as possible at the tempo of the auditory stimulus just heard while concurrently hearing mentally the stimulus itself (conditions Cl2 or Mu2). The session continued with the recordings of IWFEs in a reversed order (i.e., with the Mu conditions if Cl was recorded first, or vice versa). In order to obtain a balanced number of performances for the eight chosen reference tempi, the three tempi that were selected for the blocks were randomized for each single session.

### Kinesio® Tex Tape application

The Kinesio® Tex Tape is comprised of a polymer elastic strand wrapped by 100% cotton fibers. It allow for a longitudinal stretch of 55–60% of its resting length. The Kinesio® Tex Tape is applied to the paper substrate with 25% of tension; the adhesive is 100% acrylic (Kase et al., 2013). For the KTT (Kinesio® Tex Gold™ FP—2″ Red) application the subject, already seated on the chair with own right forearm rested on the armrest, was asked to keep the wrist in a position of maximum flexion, and the distance between the lateral epicondyle of the humerus and the distal end of the third phalanx of the middle finger was measured. The purpose was to apply the tape over the open kinetic chain including wrist, metacarpal, and finger joints. During the application of KTT, between the lateral epicondyle of the humerus and phalanges, the wrist and forearm were maintained in full flexion and full pronation, respectively (Figure 1C). After manually assessing the origin and insertion of muscles, KTT was applied over the extensor carpi ulnaris, extensor carpi radialis longus and brevis, extensor digitorum, extensor indicis, extensor digiti minimi, and extensor pollicis longus (Figures 1D–F). The strip of Kinesio® Tex was cut 5 cm longer than the maximum length of the kinetic chain measured with the wrist in maximum flexion (Kase et al., 2013). The course of the tendons of the extensor muscles (for each finger) was then identified on the back of subject's hand and distances were measured between the distal end of each phalanx and the wrist. Measurements were used to cut the distal side of the elastic band into five branches to be placed over the metacarpal area and fingers following the course of the tendons. KTT was then applied from 5 cm proximal to muscles insertion to facilitate muscle function; the application tension was light, about 25%. From the lateral humeral epicondyle, KTT was applied—in a wave-like pattern—to wrap the wrist's extensors until reaching the wrist. Each branch was then applied down on the back of phalanx until the nail.

### Statistical analysis

To model directly the observed IWFEs durations we adopted random effect ANOVA models for repeated measurements (Pinheiro and Bates, 2000; Diggle et al., 2002). Separate models were adopted for different kinds of conditions and also the response variable was chosen differently according to the presence of audio. In particular, the differences between observed and the expected IWFEs durations were considered as response variable for performances under audio (Cl and Mu) and recall (Cl2 and Mu2) conditions. This response variable measures the error observed in the IWFEs durations, the observed IWFEs duration was instead considered as the response variable for performances in the Fr condition. As the response variables have been recorded several times for each performance and for each individual, a random effect part had to be included to take into account the lack of independence among the observations. Typically, in the random effect ANOVA models adopted for the analyses, the main effects concern the effect of the overall mean of the absence/presence of KTT (N/KTT vs. KTT) and of the tempo (fast if <517.33 ms, slow otherwise). The random effect part of the ANOVA models was specified in order to separately measure the variability within individuals and within performances. Moreover, the residual variance among observations, once taken into account for the main fixed and the random effects, was further modeled to take into account possible residual heteroscedasticity.

Moreover, in order to evaluate the precision of IWFEs, we used coefficients of variation (CVs = standard deviation/mean × 100). CVs of IWFEs durations were used to investigate whether the KTT influences the precision of IWFEs performances under different conditions and, if not indicated otherwise, are to be considered as the condition's and the range's means.

Lag-one autocorrelation analysis is used as statistical signature to investigate the processes for temporal regulation. It is assumed (see Wing and Kristofferson, 1973a,b) that series of produced time intervals regulated by the event-based process should have negative lag-one autocorrelation values (between −0.5 and 0). Conversely, emergent timing is characterized by positive lag-one autocorrelation values (between 0 and 0.5). To study the influence of KTT in the Cl and Cl2 conditions we computed series of windowed lag-one autocorrelations wγ(1) (Delignières and Torre, 2011) for each set of IWFEs. Each windowed autocorrelation coefficient was computed as the mean of a set of 30 autocorrelations. Moving the set along the sequence, a series of windowed autocorrelation coefficients was computed for each performance, measuring autoregressive linear dependency within the IWFEs. Then, we calculated the mean of the obtained windowed lag series for each performance. We considered the mean wγ(1) as an estimator of overall dependence in the performance. Furthermore, we calculated the percentage of positive and negative windowed wγ(1) values for each performance. If not indicated otherwise, the mean of wγ(1) and the percentage of positive and negative wγ(1) are to be considered as the condition's and the range's means. To analyze the observed wγ(1) we adopted random effects ANOVA model for repeated measurements. In order to allow an appropriate use of parametric statistical tests, the Fisher's Z-transformation was used to normalize the distribution of autocorrelation coefficients (Nolte et al., 2004; Freyer et al., 2012).

## Results

### Timing variability

**Figure 2:**
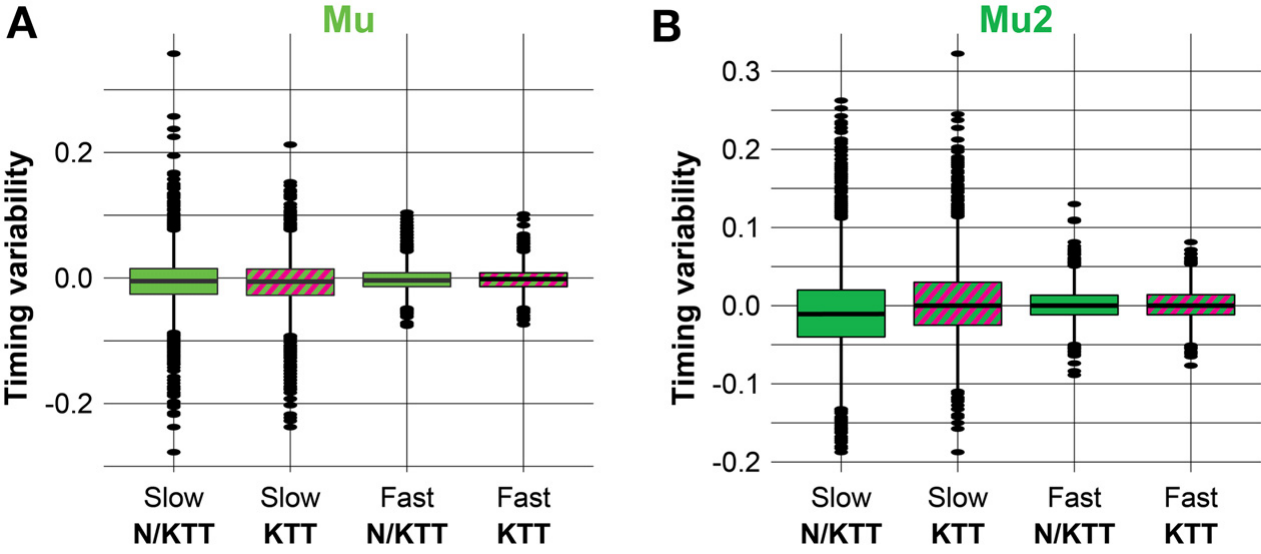
**(A,B)** Box plots of the response variable, i.e., the difference between the observed and the expected interval durations, for datasets in the conditions Mu and Mu2, respectively. Pairs of N/KTT—KTT box plots are noticeably similar when compared for slow (≥517.33 ms) vs. fast (<517.33 ms) IWFEs durations. Also, easily appreciable is that the median, the upper and lower hinges and the whiskers are very similar for pairs of N/KTT—KTT parallel box plots in slow and fast collections of IWFEs durations. Conversely, the queues of the distributions are reduced when KTT is applied (the only exception is a single far out value in the condition Mu2 for slow IWFEs with KTT). KTT reduces the variability of IWFEs durations.

Examples of our datasets are illustrated in Figure 2 as parallel box plots, for the Mu and Mu2 conditions. It is interesting to note that pairs of N/KTT—KTT plots are relatively analogous when compared for slow (≥517.33 ms) or fast (<517.33 ms) movements durations. Also, it is easily appreciable that while median, upper and lower hinges and whiskers are visibly similar for pairs of N/KTT—KTT parallel box plots in the slow and fast collections of movements durations, the position and especially number of outside values is reduced when KTT is applied, with the lonely exception of a single far out value in the condition Mu2 for slow movements with KTT.

We estimated separate random effect ANOVA models for the different conditions: without audio information (Fr), with clicks (Cl), or music (Mu), and in the recall conditions (Cl2 and Mu2). The estimates for the main effects for the models in the two audio conditions Cl and Mu are reported in Table 2, together with their *p*-values and confidence intervals. As aforementioned, for these models the response variable is the difference between the observed and the expected IWFEs durations. As can be seen in Table 2, the overall mean is negative and significant in both models. This implies that, under a slow stimulus, observed IWFEs durations are, on mean, shorter than expected. The effect of tempo (movement duration) is significant in both Cl and Mu conditions, correcting the mean error toward zero. Conversely, KTT has no significant effect on the mean error.

**Table 2:** **Estimate, *p*-values (in parenthesis) and confidence intervals for model parameters in random effects ANOVA models for the Cl and Mu conditions**.

	**Cl**		**Mu**	
**Main fixed effects**	**Estimate (*p*-value)**	**95% confidence interval**	**Estimate (*p*-value)**	**95% confidence interval**
Overall mean	−0.0063 (0.0000)	−0.0086; −0.0039	−0.0056 (0.0100)	−0.0099; −0.0013
Movement duration	0.0021 (0.0283)	0.0002; 0.0040	0.0042 (0.0401)	0.0002; 0.0083
N/KTT vs. KTT	−0.0009 (0.3349)	−0.0009; 0.0026	−0.0020 (0.3004)	−0.0058; 0.0018
**Random effects**	**Estimate**	**95% confidence interval**	**Estimate**	**95% confidence interval**
Within-individual SD	0.0045	0.0032; 0.0064	0.0066	0.0042; 0.0101
Within-set SD	0.0049	0.0042; 0.0057	0.0115	0.0101; 0.131
KTT residual variance ratio	0.8635	0.8440; 0.8835	0.9242	0.9036; 0.9452

The parameters concerning the random effects are also of interest. These parameters specifically shape the variability of the response variable. All the parameters for the random effects reported in Table 2 are significant according to the appropriate likelihood ratio test. Remarkably, the most relevant result concerns the fact that the residual variability resulted heterogeneous between N/KTT or KTT cases. The KTT-specific residual variances (usually called factor-specific residual variances, see Muthén, 1989; for details on random effects model for heterogeneous population) result significantly different (Table 3). Table 3 reports the estimate of the ratio between the residual variance for N/KTT and KTT cases. Both in the case of Mu and of Cl stimuli, this ratio is significantly less than one, assessing that the presence of the tape significantly help to decrease the variability of the errors in IWFEs durations. In particular, residual variance for KTT cases is about 14% less of the variance for N/KTT cases in Cl and 8% less in Mu.

**Table 3:** **Factor specific residual variances (σ^2^) for N/KTT vs. KTT movements**.

**Condition**	**N/KTT (σ^**2**^)**	**KTT (σ^**2**^)**	**N/KTT vs. KTT (*p*-value)**
Cl	0.00076729	0.0006625549	<0.0001
Mu	0.000772	0.000714	<0.0001
Recalls (Cl2 and Mu2)	0.00085849	0.0008080108	<0.0001
Fr	0.00082369	0.0007996383	0.005

A similar model was adopted for the recalls Cl2 and Mu2, recorded 2 min after the audio stimuli (Table 4). In this second model, the main effects are the overall mean, the condition and the movement duration, and the absence or presence of KTT. None of the estimates are significant, suggesting that, on mean, there is no evidence of a systematic error in IWFEs durations when movements are performed during recalls of music or clicks, recalls of fast or slow audio stimuli, and recalls with N/KTT or KTT. Interestingly, the presence of KTT still helps to significantly reduce the residual variance of about 6% (Table 3). The KTT residual variance ratio is estimated 0.9412 (95% confidence interval: 0.9252; 0.9575).

**Table 4:** **Estimate, *p*-values (in parenthesis) and confidence intervals for model parameters in random effects ANOVA models for the Recalls and Fr conditions**.

	**Recalls (Cl2 and Mu2)**		**Fr**	
**Main fixed effects**	**Estimate (*p*-value)**	**95% confidence interval**	**Estimate (*p*-value)**	**95% confidence interval**
Overall mean	−0.0014 (0.7492)	−0.0071; −0.0099	0.7218 (0.0000)	0.6865; 0.7571
Movement duration	−0.0034 (0.4347)	−0.0121; 0.0052	−0.3147 (0.0000)	−0.3571; −0.2723
N/KTT vs. KTT	0.0031 (0.4836)	−0.0055; 0.0117	0.0114 (0.5934)	−0.0306; 0.0533
**Random effects**	**Estimate**	**95% confidence interval**	**Estimate**	**95% confidence interval**
Within-individual SD	0.0031	1e-04; 0.0993	0.0127	1e-04; 1.2508
Within-set SD	0.0377	0.0347; 0.0410	0.1452	0.1306; 0.1614
KTT residual variance ratio	0.9412	0.9252; 0.9575	0.9708	0.9511; 0.9908

For the condition without audio stimulus (Fr), the model considers as the response variable directly the observed duration (Table 4). Consequently, the overall mean simply measures the mean movement duration chosen for performances of slow movements with N/KTT, while the main effect of movement duration measures the increment in bpm implied by a faster tempo. The overall mean and the main effect of tempo are then obviously significant by construction. The main effect of KTT is not significant. For this particular model, this result implies that individuals choose the tempo of the performance independently of the presence of the tape on their arm. Regarding the random part of the model, again it is worth to notice that the effect of the KTT in reducing the residual variability is still significant (Table 3). The residual variance, small but still present, is about the 3% less in KTT cases (estimate: 0.9708; 95% confidence interval: 0.9511; 0.9908).

**Figure 3:**
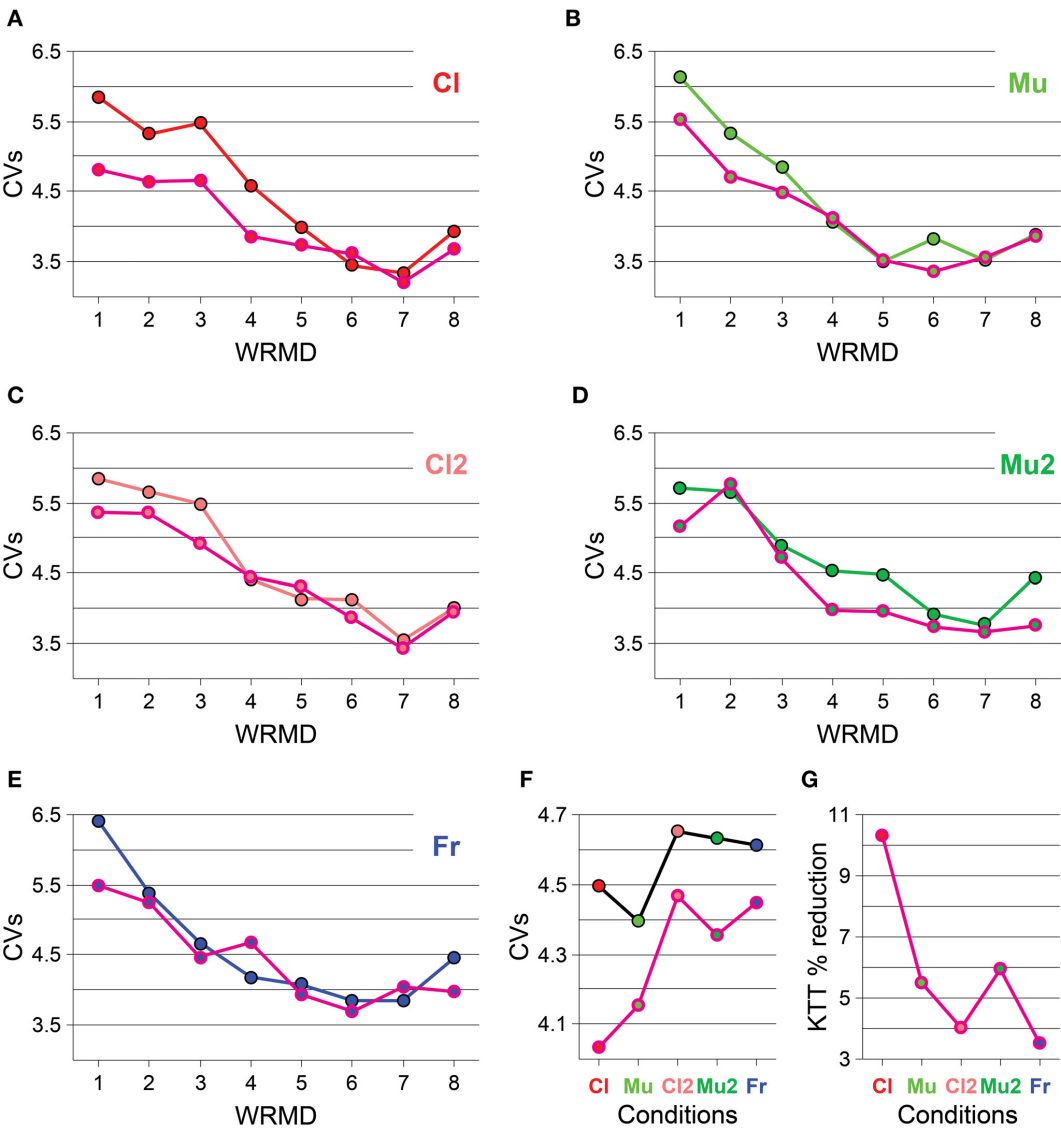
**(A–E)** For each condition the CVs of IWFEs durations, ranked per within-ranges of durations, are illustrated. **(F)** The five within-condition CVs of IWFEs durations are compared for N/KTT and KTT. **(G)** The percent reduction of the within-condition CVs values when KTT is applied. The reduction is more than 3% in all conditions; the most evident reduction, more than 10%, is for the Cl condition. Values are plotted as black (N/KTT) and pink (KTT) outlined circles, filled-in color-coded per condition. Color-coded connecting lines are shown to ease reading. CVs = within-range or, in **(F)**, within-condition CVs values. WRMD, within-ranges movements durations.

The CV of IWFEs is used to normalize measures of temporal variability. We plotted the CVs of IWFEs as a function of the ranges of movement duration to visualize the rate dependent changes of the precision of isochronous performances and the interactions between these changes and the different experimental cases (N/KTT or KTT). The CVs of IWFEs, ranked per within-ranges of IWFEs durations, are illustrated in Figure 3 for all conditions (Figures 3A–E). CVs values appear to change with movement duration. In particular, CVs of IWFEs become smaller as IWFEs become faster and, within the same condition and for the same range of movement duration, CVs values are very often smaller in KTT than in N/KTT cases. In Figure 3F the five within-condition CVs of IWFEs durations are compared for N/KTT and KTT cases. The CVs values of IWFEs are below 5% in all conditions and for both N/KTT and KTT cases. It is also noticeable that the within-condition CVs values are always smaller in KTT, when values for the same condition are compared, and that the difference is maximal for the condition Cl. Also, in Figure 3G are shown the percent reductions for the within-condition CVs in the case of KTT application. The percent reduction for the within-condition CVs values is maximal when IWFEs are performed while listening to an audio stimulus such as clicks (10.33%, for Cl). Impressively, a percent reduction for within-condition CVs values in KTT is visible in all other conditions (5.47%, for Mu; 4.03%, for Cl2; 5.96%, for Mu2; and 3.50%, for Fr; see Figure 3G).

### The processes for temporal regulation

To explore whether, and to what extent, the processes for temporal regulation are influenced by the application of KTT, the mean wγ(1) values and the percentages of positive wγ(1) of IWFEs computed in N/KTT and KTT cases are compared. We plot the mean wγ(1) values and the percentages of positive wγ(1) as function of ranges of movement durations of IWFEs. As an exemplar comparison, we here describe and analyze the Cl and Cl2 conditions since we already showed them to be the most subjective to the application of KTT.

**Figure 4:**
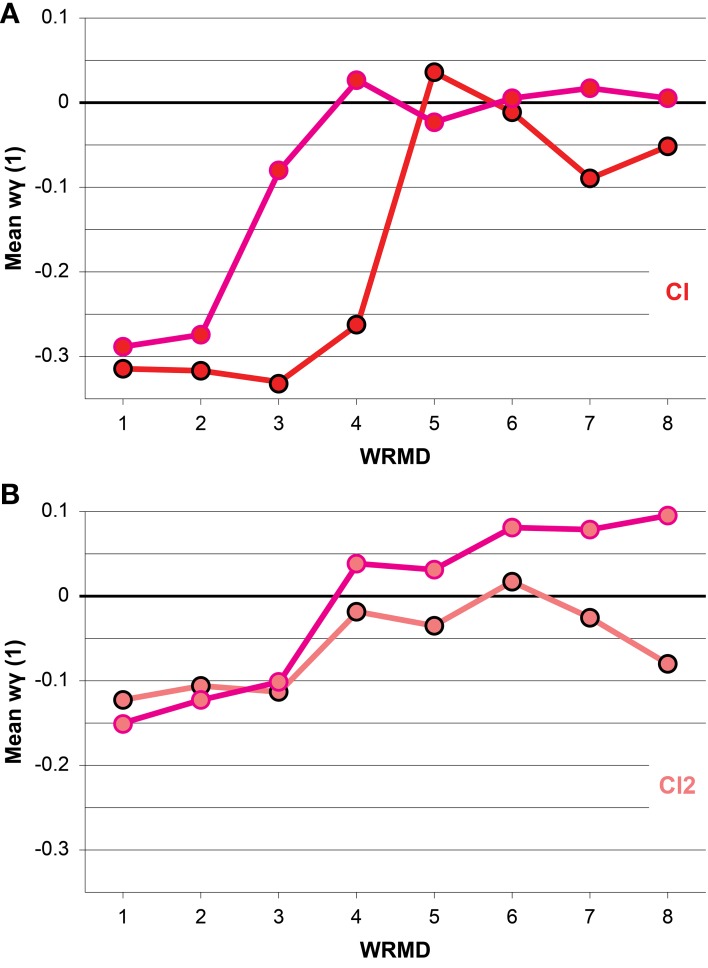
**(A,B)** Mean windowed wγ(1) values, ranked per within-ranges of durations, are illustrated. The conditions Cl and Cl2 are compared for N/KTT and KTT. Note that mean wγ(1) values appear to change with movement durations and are, for both Cl and Cl2 conditions, biased toward more positive values when KTT is applied. Values are plotted as black (N/KTT) and pink (KTT) outlined circles, filled-in color-coded per condition. WRMD, within-ranges movements durations.

The mean wγ(1) values of IWFEs ranked per within-ranges of movement durations are displayed in Figure 4 for the N/KTT and KTT cases of the Cl and Cl2 conditions. In all cases mean wγ(1) values appear to change with movement durations. In the N/KTT case of the Cl condition (Figure 4A) mean wγ(1) values are almost all negative with only one exception in range 5 (for this and the following references to ranges of movement durations see Table 1) whereas in the KTT case of the Cl condition (Figure 4A) mean wγ(1) values are negative for only the half of eight ranges of movement durations. The highest peaks of mean wγ(1) values are expressed within the moderato, allegro and presto tempi in the last four ranges of interval durations (Cl of N/KTT) and in the last five ranges of movement durations (Cl of KTT).

In the N/KTT case of the Cl2 condition (Figure 4B) mean wγ(1) values are all negative with only one exception in range 6 whereas in the KTT case of the Cl2 condition (Figure 4B) mean wγ(1) values are positive for more than half of ranges of movement durations. The highest peaks of mean wγ(1) values for the Cl2 condition are expressed within the moderato, allegro and presto tempi in the last five ranges of movement durations in both N/KTT and KTT cases.

**Figure 5:**
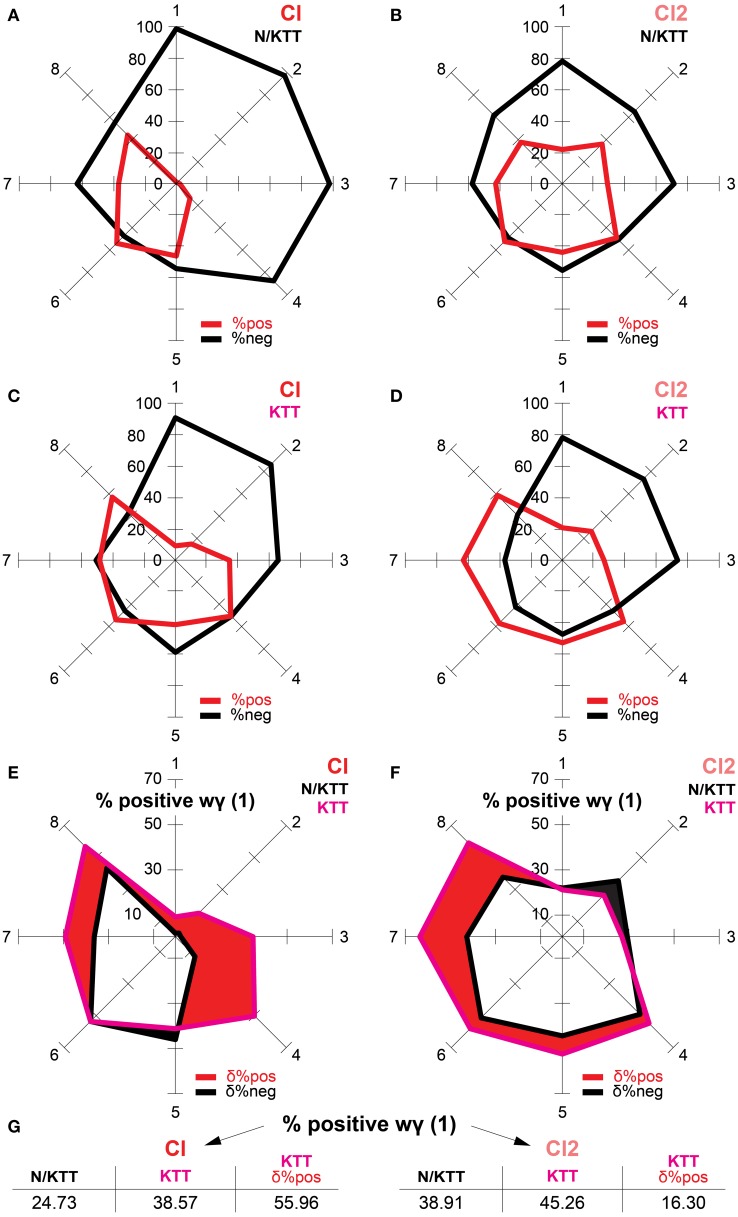
**(A–D)** On the radar charts are illustrated, for the N/KTT and KTT cases of Cl and Cl2 conditions, the percentages of positive and negative wγ(1) values ranked per within-ranges of durations. Note that the areas generated by the eight within-range percentages of positive wγ(1) values are wider in the KTT than in N/KTT cases for both Cl and Cl2 conditions. **(E,F)** The percentages of N/KTT vs. KTT positive wγ(1) values are compared for the Cl and the Cl2 conditions, respectively. The red area indicates for each of the eight within-ranges of durations the differential percent of positive wγ(1) values. This area is wider in the KTT than in the N/KTT case, both for the Cl and the Cl2 conditions. Compound shapes are obtained by combination of areas of positive wγ(1) values in the N/KTT and KTT cases for both the Cl and Cl2 conditions. The overlapping area was excluded, turning the filled region into a hole. **(G)** The increase of positive wγ(1) values in the KTT case is comprehensively indicated as differential percent equivalent both for the Cl and Cl2 conditions (see text for more explanations). %pos (red) = percentages of positive wγ(1) values. %neg (black) = percentages of negative wγ(1) values. δ%pos (red) = differential percent equivalent for percentages of positive wγ(1) values. δ%neg (black) = differential percent equivalent for percentages of negative wγ(1) values.

The percentages of positive and negative wγ(1) values ranked per within-ranges of movement durations are displayed in Figure 5 as radar charts for the N/KTT and KTT cases of the Cl and Cl2 conditions. Here also, it appears evident that the percentage of positive wγ(1) values is sensitive to movement durations.

The values of wγ(1) of IWFEs in the Cl condition are mostly negative for the N/KTT case (75.27% of negative values and 24.73% of positive values). In the KTT case, although the amount of positive values increases, negative wγ(1) values continue to outnumber positive ones (61.43% of negative values and 38.57% of positive values). In the N/KTT case of the Cl condition (Figure 5A), percentages of positive wγ(1) values are extremely low in the first four ranges of movement durations. The highest peaks for percentages of positive wγ(1) are evident in the last four ranges of movement durations. The highest peaks for percentages of positive wγ(1) values are always less than 50%, with only one exception (range 6, 53.23%). In the KTT case of the Cl condition (Figure 5C), percentages of positive wγ(1) values are extremely low only in the first two ranges of movement durations. However, the percentages of positive wγ(1) values in the two successive ranges (3 and 4) increase from the 2.19 and 12.38% in the N/KTT case to the 34.38 and 49.93% in the KTT case, respectively. Also, in the last four ranges of movement durations of KTT case, the percentages of positive wγ(1) values are similar to those in N/KTT case.

The values of wγ(1) of IWFEs in the Cl2 condition are somewhat negative for the N/KTT case (61.09% of negative values and 38.91% of positive values) while in the KTT case negative wγ(1) values slightly outnumber positive ones (54.74% of negative values and 45.26% of positive values). In the N/KTT case of the Cl2 condition (Figure 5B), percentages of positive wγ(1) reach lowest values in the first and third ranges of movement durations. In the KTT case of the Cl2 condition (Figure 5D), percentages of positive wγ(1) reach extremely low values only in the first three ranges of movement durations. The highest peaks for percentages of positive wγ(1) values are in the five last ranges of movement durations both in the N/KTT and KTT case. It is also well perceivable that while in the N/KTT case only one of the within-range percentage of positive wγ(1) values is above the 50%, in the KTT case five of the eight within-range percentages of positive wγ(1) values are above the 50%.

In Figures 5E,F the percentages of positive wγ(1) values of N/KTT vs. KTT cases are compared separately for the Cl and the Cl2 conditions. As illustrated, the area generated by eight ranges percentages of positive wγ(1) values is wider in the KTT than in the N/KTT case, both for the Cl and the Cl2 conditions. For the Cl condition, the differential percent of positive wγ(1) values is of 13.84% (calculated as difference between the percentages of positive wγ(1) of 38.57% in KTT case and that of 24.73% in N/KTT case). This differential percent is equivalent to an increase of 55.96% of positive wγ(1) values in the KTT case (Figure 5G). Similarly, for the Cl2 condition, the differential percent of positive wγ(1) values is of 6.34% (calculated as difference between the percentages of positive wγ(1) of 45.26% in the KTT case and that of 38.91% in the N/KTT case). In the Cl2 condition, the differential percent is equivalent to an increase of 16.30% of positive wγ(1) values in the KTT case.

Finally, random effect ANOVA model for repeated measurements is performed for the wγ(1) values in Cl and Cl2 conditions, respectively. The model for Cl reveals a highly significant effect for the presence of KTT [*F*_(1, 98)_ = 15.126, *p* < 0.001] and tempo [*F*_(1, 98)_ = 59.62, *p* < 0.001]. The interaction between the presence of KTT and tempo shows also a significant effect [*F*_(1, 98)_ = 5.05, *p* < 0.05]. The model for Cl2 fails to reveal a significant effect of presence of KTT, [*F*_(1, 101)_ = 0.34, *p* > 0.05] and tempo, [*F*_(1, 101)_ = 3.69, *p* > 0.05]. Conversely, the interaction between the presence of KTT and tempo shows a significant effect [*F*_(1, 101)_ = 5.05, *p* < 0.05]. It should be remembered here that in the Cl2 condition mean within-range wγ(1) values become more positive in KTT than in N/KTT only for fast tempi (see Figure 4B).

## Discussion

In this study, we examined whether the application of KTT on skin is able to reduce the timing variability of repetitive rhythmic movements in healthy subjects.

Overall, our results indicate that the temporal variability of IWFEs is influenced by the application of KTT. Using a task of auditory-motor integration recently developed in our laboratory (see Bravi et al., 2014) we demonstrate that the timing variability is reduced in the KTT case, independently of the type of sensorimotor integration required from the participant to accomplish the motor performance (i.e., without audio information, with paced audio in the form of clicks or music, and in the recall conditions). Quantitatively, the reduction in the timing variability is different for the different conditions, and is greatest for the Cl and smallest for the Fr conditions. However, even though the reduction in timing variability for the Fr condition is quantitatively smaller than those estimated for the other conditions, the residual variance for KTT case is about 3% less than the variance of N/KTT case, which is of great importance. In fact, the residual variance evaluates the variability in the IWFEs durations when subjects are following their own tempo, which is mostly linked to the physical performance of the movement, without the effort of following or recalling a tempo proposed by others. Also, our analysis of the CVs of IWFEs show that, in general, when IWFEs are performed in presence of KTT the within-condition CVs values are smaller than those obtained in the N/KTT case. Here, again, the reduction in the CVs value is maximal when IWFEs are performed while listening to an audio stimulus such as clicks and minimal when performed in the Fr condition.

Our first experimental hypothesis is that KTT brings about an improvement of wrist joint proprioception due to augmented afferent input via the stimulation of cutaneous mechanoreceptors. This effect would, in turn, augment the coordination of the wrist joint during the rhythmic motor performance and consequently contribute to the reduction in timing variability of the IWFEs. The type of KTT application that we used allowed us to speculate about this possible effect provided by KTT: indeed, since the elastic band, placed between the lateral epicondyle of the humerus and the phalanges, is applied with the wrist positioned in maximum flexion, it cannot provide a facilitation of the ascent phase of the movement by means of elastic return to the starting position. This consents us to discard the possibility that the reduction of timing variability in the KTT condition is due to the dampening of gravity, but leads us toward an explanation that KTT might enhance proprioceptive information provided by activating cutaneous receptors.

Proprioception consists of a combination of joint position sense, i.e., the ability to sense the position and movement of a limb in space (Aydin et al., 2001), and the sense of muscular effort and tension (Proske and Gandevia, 2009). The joint position sense is a very important contributor to joint coordination, maintenance of muscle stiffness, and to the production of natural movements for appropriate task performance (Han and Lee, 2014). It was demonstrated that the principal muscle receptor in joint position sense is the muscle spindle; however, also cutaneous receptors have become recognized as playing an important role (Proske and Gandevia, 2009). The cutaneous receptors, subserving a sense of position and movement, respond to the stretching of skin (Proske and Gandevia, 2009). As proposed by Grigg (1994), it is plausible that the application of KTT induces, during the joint movement, a pressure and a stretching/deformation of the skin, thus activating cutaneous mechanoreceptors. Therefore, the mechanical effects of KTT applied to skin, augmenting skin receptor output, might enhance kinesthetic and joint position sense (Simoneau et al., 1997; Halseth et al., 2004).

Zelaznik and Rosenbaum (2010) performed an analysis in which timing precision was measured at different locations during both the performance of tapping and circle drawing tasks. Evidences showed that the highest values of CVs during the performance of the tapping task were at a location opposite to the specified timing location, that is, the maximum of the extension point. Earlier, Semjen and Garcia-Colera (1986) also noted that, in a tapping motor performance, timing variability showed smallest values at the instructed timing location. Hence, in tapping tasks the subject is timed to a location and the timing variability is best controlled at the corresponding event (e.g., the contact with the tap key); in circle drawing the subject is not timed to a place (i.e., is not required to rely on a target location or event) but is controlling evenly the entire movement trajectory (Spencer and Zelaznik, 2003). Our experimental design is not based on a tapping task, although the IWFEs, having clear turning points that provide salient sensory information (Elliott et al., 2009), are fairly similar. The KTT seemingly relieves, at least in part, from the need of a target or an event inherent with the kinematics of IWFEs. This is particularly evident in the Cl (see Figure 3) but also in the Mu audio conditions in which movements are performed with additional sensory cues. The extra proprioceptive information provided by KTT, augmenting the stability of wrist joint during the performance, could very well account for the reduction in IWFEs temporal variability that we observed as being more pronounced in our cued conditions.

In addition, we explore the possible influences of our KTT on the neural processes governing the temporal regulation for production of rhythmic movements. The mean wγ(1) values and the percentages of positive wγ(1) of IWFEs, calculated in N/KTT and KTT cases, and compared for the exemplar Cl and Cl2 conditions, show that the processes for temporal regulation can be influenced by the application of KTT. We demonstrate that the mean wγ(1) and percentages of positive wγ(1) are in fact biased toward values congruent with the emergent mode for control of timing.

Overall, these results have important implications for the event-emergent timing distinction. Heretofore, it was suggested that a task is controlled via event timing or via emergent timing depending on the kinematic of the performed movement (Zelaznik and Rosenbaum, 2010). Huys et al. (2008) using a rhythmic motor task, such as tapping, have demonstrated that discrete and continuous movements are two classes of movements topologically distinct. Indeed, it was shown that when finger flexion-extensions are performed as slow and discrete movements, the engagement of an explicit timing (event-based) process is required; whereas when the tapping is performed as rapid and smooth movements, the employment of a self-organized limit-cycle (emergent) process is necessary. The clarity with which such events are delineated in a particular experimental situation is considered to be the factor determining the weight of the event-based timing component. The continuity of the movement largely determines the strength of emergent timing component (Repp and Steinman, 2010). In addition, in a timing study on air finger tapping, participants performed discrete movements, being instructed to pause before each flexion cycle, and continuous movements, being instructed to move as smoothly as possible without pausing (Spencer et al., 2007). Despite the subtle difference between these discrete and continuous movements, activation in the cerebellum was greater when participants were instructed to perform discrete movements, suggesting that the engagement of the cerebellum may depend on how movements are produced or, even better, on how they are represented (Spencer et al., 2007).

Our experimental hypothesis is that the application of KTT, by stabilization of the motor effector during the performance, causes the IWFEs to be less discrete and thus more continuous. According to the dichotomic view of the event-based vs. emergent modes of temporal control (Delignières and Torre, 2011), KTT could promote the involvement of emergent timing control and, consequently, reduce the contribution of event-based timing. As a consequence, the control for timing would no longer need to refer to a nervous structure such as, for example, the cerebellum, in the building of an abstract representation of the time intervals to produce. Rather, the dynamics of the system could be sufficient *per se* to keep the movement cycle constant.

We demonstrate a reduction in timing variability with KTT, as seen by the CVs values and their dispersion, being very often smaller in the KTT than in the N/KTT cases (see Figures 2, 3). Such reduction seemingly suggests a transition toward an emergent timing control. This emergence of the emergent timing strategy (see Figures 4, 5E–G) appears to be by virtue of the KTT's to render movements less audio sensitive or dependent (compare, in Figures 5A–C vs. B–D). This mechanism ultimately results in a reduced variability and thus, in a better—more homogenous—performance. The same concept is analogous to a computer that allocates memory for a better performance; so do the cortical and subcortical circuits, which are usually required to bear the load of motor control and of other cognitive activities. Being less dependent on external/discrete events, central structures are partially relieved from timing control, thus freeing resources and allowing for a net augmentation of the central efficiency for motor control and cognition.

In conclusion, KTT was used in this study as a tool to possibly augment the precision of rhythmic movements. Our results show that KTT does, in fact, reduce the timing variability of rhythmic movements performed with no direct surface opposition and minimizing visual information. In particular, our results suggest that KTT—on the one hand by providing extra proprioceptive information, and on the other hand by relieving, at least partially, the central structures from time control—allows for a net augmentation of the central efficiency for motor control.

## Author contributions

Riccardo Bravi: Substantial contributions to the conception of the work, the data acquisition, analysis, and the interpretation of data; Substantial contribution in drafting the work and revising it critically for important intellectual content; final approval of the version to be published; agreement to be accountable for accuracy and integrity of any part of the work. Eros Quarta: Substantial contributions to the design of the work and data acquisition; Substantial contribution in drafting the work; final approval of the version to be published; agreement to be accountable for accuracy and integrity of any part of the work. Erez J. Cohen: Substantial contributions to the design and the data acquisition of the work; Substantial contribution in drafting the work; final approval of the version to be published; agreement to be accountable for accuracy and integrity of any part of the work. Anna Gottard: Substantial contributions to the analysis and interpretation of data for the work; Substantial contribution in drafting the work; final approval of the version to be published; agreement to be accountable for accuracy and integrity of any part of the work. Diego Minciacchi: Substantial contributions to the conception of the work, analysis, and interpretation of data for the work; Substantial contribution in revising the work critically for important intellectual content; final approval of the version to be published; agreement to be accountable for accuracy and integrity of any part of the work.

### Conflict of interest statement

The authors declare that the research was conducted in the absence of any commercial or financial relationships that could be construed as a potential conflict of interest.

## Abbreviations

bpm: beats per minute
Cl: while listening to a stream of clicks
Cl2: after 2 min from the end of the Cl condition
CVs: coefficients of variation
Fr: free, in absence of auditory information
IWFEs: isochronous wrist's flexion-extensions
KTT: Kinesio® Tex taping
Mu: while listening to an excerpt of music
Mu2: after 2 min from the end of the Mu condition
N/KTT: no KTT
wγ(1): windowed lag-one autocorrelations.
